# The gut-masticatory muscles-temporomandibular joint pain axis—a scoping review

**DOI:** 10.22514/jofph.2025.021

**Published:** 2025-03-12

**Authors:** Gayathri Krishnamoorthy, Aparna Narayana, Dhanasekar Balakrishnan

**Affiliations:** ^1^Department of Prosthodontics and Crown Bridge, Manipal College of Dental Sciences, Manipal Academy of Higher Education, 576104 Manipal, India

**Keywords:** Orofacial pain, Temporomandibular joint, Microbiome, Probiotics, Brain-gut axis

## Abstract

Orofacial pain has become the most common debilitating disease resulting in high healthcare costs, and compromising the quality of life, speech, aesthetics and masticatory function of those affected. As its aetiology is multifactorial and as the treatment involves a multidisciplinary holistic approach, arriving at a confirmative diagnosis is challenging. Numerous studies have been published that support the bidirectional link between gut health and other organs like the cardiovascular system, respiratory system, neurological and hormonal. Recent studies indicate a potential link between gut microbiota dysbiosis and chronic orofacial and temporomandibular joint (TMJ) pain. In this review, we enumerate the link between the metabolites released by the gut bacteria and how they regulate the pain mechanism of various types of orofacial pain like chronic, neuropathic and inflammatory in the orofacial and TMJ regions. We also discuss the potential link between pain and gender predisposition. Further, we review the recent non-invasive therapeutic options which can be put forth to use for treating orofacial and TMJ pain.

## 1. Introduction

The entire microbiota in the human body, also called the “microbiome”, outnumber the total cells in the human body. Out of the total microbiota in the human body, almost 95% of the microbiome resides in the colon [1]. Since 2007 after the inception of the Human Microbiome Initiative funded by The National Institutes of Health (NIH), novel discoveries on the interlink between gut microbiota (GM) and human diseases have been made [2]. These disorders associated with abnormal microbiome are called dysbiosis.

The gut microbiome influences the human body through 3 major pathways namely the neural route, the immune route and the hormonal route, together these constitute the gut-brain axis (GB Axis). In the last 15 years, after the development of microbiome science, massive development and attention have been given to the GB Axis and its interlink with the pathophysiology of various disorders like psychiatric, neurological and musculoskeletal [3, 4, 5, 6].

While the sympathetic afferent fibres were considered the only source of signalling mechanism and interoceptive information, microbiota science establishes that gut microbes and their metabolites also act as another prime signalling mechanism in the human body [7]. Experimental studies have proven that GM dysbiosis and metabolites produced by the GM itself affect the Central Nervous System (CNS) activity thereby modulating the pain axis and leading to chronic orofacial and temporomandibular joint (TMJ) pain [8].

There are limited studies which confirm gut dysbiosis and its interlink with chronic orofacial pain [8], migraine [9], neuropathic pain [10] and inflammatory pain [11]. However, there is no available literature to the best of our knowledge which covers the GM dysbiosis interlink with different types of orofacial and TMJ pain pathology in totality in a single review article. It is these complex multiple pathophysiologies that make it difficult to understand the interlink between GM-Orofacial & TMJ-Pain axis.

Therefore, this review aims to encompass and simplify the GM’s influence on the masticatory muscles and TMJ pain. We also elaborate on the influence of GM on different types of orofacial pain like chronic widespread orofacial pain, neuropathic pain, inflammatory pain, migraine and TMJ pain. Finally, we formulate a treatment plan strategy which targets GM dysbiosis.

## 2. Materials and methods

The search terms (“GM” OR “gut microbiome” OR “gut bacteria” OR “probiotics” OR “prebiotics”) AND (“orofacial pain”) were used to retrieve articles from Scopus, PubMed, Embase and Grey search.

The inclusion criteria were as follows—articles written only in the English Language, randomised control trials, quasi-randomized control trials, *in-vivo* and *in-vitro* (laboratory) studies, retrospective and prospective studies, cohort studies, case-control studies, Systematic reviews and meta-analyses which compare the interlink between GM and orofacial pain were included. Conference proceedings, Letters to the editor, Book reviews and Chapters were excluded from the study.

74 articles were obtained between the years 2016–2024, out of which 10 were duplicates and hence eliminated. After the initial title and abstract screening, 17 articles were included in this review (Fig. 1).

**Fig. 1.** PRISMA (Preferred Reporting Items for Systematic Reviews and Meta-Analyses) flow diagram of the study screening procedure and selection.

## 3. The link between GM and microbial metabolites—pain axis

The nociceptors initiate neuronal activation in the peripheral organs which convert any noxious stimuli, *e.g.*, inflammation, mechanical injury, heat or cold stimuli into nerve impulses and transmit these nociceptive signals to the spinal cord dorsal horn [12, 13]. The spinal nociceptive neurons through the spinothalamic and spinoparabrachial tracts project themselves into the thalamus, somatosensory cortex and anterior cingulate cortex to process the afferent and sensory components of pain [12]. Recent studies have however demonstrated that the non-neuronal cells, such as the glial cells, immune cells—macrophages and lymphocytes, tumour cells, *etc*. can also regulate pain in the Peripheral Nervous System (PNS) and Central nervous system (CNS) [13]. The following section will decode the relationship between these non-neuronal cells, especially the glial cells, which play a prime role in initiating and maintaining chronic pain transmission [14, 15, 16, 17] and their interlinking with the gut microbiome.

### 3.1 The gut-glial interlink in pain transmission

The activation of the non-neuronal glial cells along the pain circuits leads to the formation of a localised form of inflammation known as “neuroinflammation” in the PNS and CNS which results in the onset of visceral hypersensitivity [18, 19]. Neuroinflammation plays a vital role in chronic pain maintenance, and their interaction is always bi-directional. Few recent studies have reported that GM modulates glial cell maturation which aids in the transmission of pain [20].

An animal model study which was carried out to evaluate the antinociceptive effect of Photobiomodulation (PBM) therapy, Vitamin B Complex (VBM) and a combination of both concluded that both PBM and VBM alleviate the pain either alone or in combination by modulating the glial cells and cytokines expression in the spinal trigeminal nucleus of rats. The authors demonstrated that both the interventions used in the study attenuated the nociceptive responses by inhibiting the activation of the glial cell and thereby the production of glial-derived inflammatory mediators in the spinal trigeminal nucleus [21].

In an *in vivo* study performed to consider the effects of berberine on visceral hypersensitivity and activation of microglial cells, it was observed that microglial cells in the dorsal lumbar spinal cord were suppressed by berberine. In parallel to the *in vivo* study, the authors conducted another *in vitro* study to confirm whether Berberine directly affects microglial cell changes. It was proven that berberine inhibits the activation of microglial cells via the microbiota-gut-brain axis but does not have any direct effects [22].

Although currently, the available literature on the gut-glial pain axis is limited, it can be speculated that glial cells and gut-pain axis can prove to be a turning point in the field of precision medicine for pain management modalities and provide a solution to the chronic uncurable pain states.

Accumulating evidence also demonstrates the interlink between the metabolites released during gut dysbiosis and how they transmit pain via different signalling pathways and the Vagus nerve. In the following pages, the role of these GM and their metabolites in transmitting various types of orofacial pain will be discussed (Fig. 2).

**Fig. 2.** Interlink between gut health and various types of orofacial pain: the diagram depicts the interplay between gut microbiota dysbiosis and different types of orofacial pain: Neuropathic type, Inflammatory type, Chronic type, Migraine and tension type headache and pain due to night grinding. Created with BioRender.com.

### 3.2 The GM metabolites interlink with chronic orofacial pain

The most common orofacial pain disorders often encountered in a dental office are the TMJ and masticatory muscle pain, wherein the prevalence of TMJ disorders accounts for up to 31.1% of the adult population [23]. TMJ disorders have complex etiological findings and what makes the diagnosis even more difficult is that most of these findings are not directly associated with the stomatognathic system [24].

Gallotta S *et al*. [25] in their clinical trial investigated the prevalence and risk of TMJ disorders in patients with irritable bowel syndrome (IBS). They concluded their study by demonstrating a positive correlation between facial pain and abdominal pain. They also discovered that IBS patients had 3 times more risk of TMJ disorders as compared to healthy adults.

This nexus between IBS and chronic orofacial pain can be attributed to the treatment provided for IBS which includes either antibiotics or antidepressants depending on the IBS type. These medications disrupt the gut bacteria, producing metabolites like short-chain fatty acids (SCFA), serotonin, dopamine, amino acid metabolites, *etc*., which affect the activity of CNS [26, 27, 28]. This change in CNS activity modulates various types of orofacial pain including chronic, neuropathic, inflammatory and migraine [13].

The results were similar with another case-control study which was conducted to evaluate the association between chronic TMJ disorders and gastroesophageal reflux disease (GERD) [29]. GERD though affects the gastrointestinal tract; literature supports its association with teeth grinding or clenching. It was concluded in the study that symptomatic GERD is associated with painful, chronic TMJ disorder and due consideration should be provided to manage the gut symptoms [29]. The ant-acid medications for GERD treatment disrupt the GM, resulting in dysbiosis. The metabolites released during the dysbiosis, modulate the CNS activity resulting in chronic and painful type of TMJ pain [13].

### 3.3 The GM metabolites interlink with neuropathic type of orofacial pain

Zhou F *et al*. [30] in an animal study induced Chronic Constriction Injury (CCI) in mice to determine whether GM is involved in neuroinflammation. They discovered that Short Chain Fatty Acids (SCFAs) by-products of GM are involved in nerve injury-induced neuropathic pain, GM causes activation of microglial cells during neuropathic pain and expression of inflammatory markers in the hippocampus and spinal cord. They concluded that SCFAs regulate the activation of microglial cells and subsequently increase the pro-inflammatory markers in the hippocampus and spinal cord. However, the administration of antibiotics reduces the production of SCFAs and thereby inhibits the polarisation of microglial cells.

Burning mouth syndrome (BMS) an idiopathic orofacial pain with multifactorial etiopathology is characterized by a burning sensation of oral mucosa. The three elements of pain that BMS patients usually experience are nociceptive pain, neuropathic pain and nociplastic pain [31]. Although, there is little evidence regarding the interlink of GM with BMS, however, the nociplastic type of pain experienced in BMS, may be associated with the gastrointestinal symptoms as well as the Central Nervous System origin, which incriminates that the GM may be an etiopathology of nociplastic type of pain. However, further research is required to elucidate the mechanism behind the three elements of pain and their link with GM [31].

To observe this complex biological mechanism behind neuropathic pain and GM, Lan Z *et al*. [32] carried out a Mendelian Randomization (MR) approach to study the causal relation between Trigeminal neuralgia (TN) and GM. They concluded in their MR study that nine bacterial groups and metabolic pathways were significantly associated with an increased risk of TN. They also observed fifteen immune cells which were significantly associated with an increased risk of TN. These findings support the causal association between GM with TN and between GM and immune cells which mediate the pain.

### 3.4 The GM metabolites interlink with inflammatory type of orofacial pain

Periodontitis is a well-known inflammatory disease caused by a bacterial infection in dental plaque that progresses systematically and destroys periodontal tissues [33]. Periodontitis as opposed to other inflammatory diseases progresses without any pain symptoms, because of this, patients usually visit the clinician after severe destruction of periodontal tissue has occurred.

To decipher this mechanism behind the progression of periodontitis without any pain symptoms in the periodontal tissue, Murakami N *et al*. [33] executed a study on the molar teeth of mice which was tied with *Porphyromonasgingivalis* (*P. gingivalis*) inoculated ligature wires. It was concluded in this study that Butyric acid (BA) which is released in large quantities from *P. gingivalis *bacteria during the progression of periodontal disease changes the somatosensory characteristics of the periodontal tissue in a chronic inflammation state. BA signal via GPR41 and suppress the periodontal inflammatory pain in the Trigeminal Ganglion.

Another study performed to investigate the influence of GM on TMJ inflammation showed that Resveratrol (RSV) inhibits TMJ inflammation triggered by the administration of Complete Freund’s Adjuvant (CFA) and reverses the CFA-induced reduction of short-chain fatty acids and gut bacteria. Furthermore, it was also proved that RSV crosses the blood-brain barrier and inhibits the activation of microglial cells. Hence, the authors concluded in their study that GM dysbiosis is critical for developing TMJ inflammation and recovering the gut microbiome to its normal levels can be a new therapeutic strategy for treating such chronic inflammatory pain [34].

### 3.5 The GM metabolites interlink with migraine headaches

Migraine and tension-type headaches (TTH) are the most common forms of primary headaches affecting individuals of all age groups but typically peaking in adult populations. There has been a 16% increase in migraine cases globally in 2019 from 1990 with significant demographic variation and is distinctly elevated in women [35]. The most prevalent causative factors for migraine and TTH encompass alcohol, coffee, fatigue or stress and the usual symptoms associated are photophobia, phonophobia and gastrointestinal disturbances like nausea, vomiting, acid reflux and diarrhoea.

In a 1992 population study by Jones and Lydeard *et al*. [36] on irritable bowel syndrome (IBS) patients, 32% complained of migraine headaches as compared to the rest of the 18% of the control group. Another similar prospective study was conducted in 2004 [37] which reported 17% of IBS patients complaining of migraines as compared with the 8% control group.

Peatfield *et al*. [38] in their survey among migraine patients observed that specific foods were reported as a trigger factor for their migraine-induced headaches. Among these, 19% of them reported chocolate as a trigger factor, 18% reported cheeses and 11% reported citrus fruits as a cause of their migraine.

These are some of the many articles which have disproved an interlink between gut health and migraine [39, 40, 41], however, it was still unclear how an alteration in the GM and their metabolites affect migraine headaches.

It was Tang Y *et al*. [9] that induced migraine-like pain in mice using nitroglycerine (NTG) to study the underlying mechanism behind the interlink between headache and GM. They also investigated the involvement of tumour necrosis factor-alpha (TNF-α) in migraine-type headaches as previous studies had shown that NTG administration in rodents caused light aversive behaviours and an increase in TNF-α [42, 43, 44, 45], which was also found higher in migraineurs. It was concluded in their study that antibiotic treatment prolonged NTG-induced acute migraine-like pain. Furthermore, on the genetic deletion of TNF-α or injection of TNF-α antagonist into intra-spinal trigeminal nucleus caudalis, the pain prolongation was completely blocked. On faecal microbiota transfer, the colonisation of the gut microbiome was reversed and alleviated pain.

Hence, the results of their study indicate a direct interlink between GM dysbiosis and migraine-like pain and recovering the gut microbiomes to a healthy state, does bring about alleviation of pain. Consequently, the alteration of the GM can be used as a new therapeutic option for treating migraine-like pain.

## 4. Gender predisposition-GM-pain axis

Among the host of factors which affect gut health, gender and sex hormones play an important role post-puberty. Surprisingly, the bacteria-to-human cell ratio is higher in women than men, *i.e.*, 2.2 in women and 1.3 in men [46]. As age advances, there is a constant change in the composition of GM from childhood to old age, due to changes in diet, lifestyle, environmental factors, medication, *etc*. Few studies that have focused on this field of research, “Microgenderome”, suggest that sex hormones bring about changes in GM [47, 48, 49]. However, recent studies are focusing on the differences in pain sensitivity between genders and their association with gut microbiota composition.

Literature suggests consistent differences in pain physiology between men and women, with women presenting more pain conditions than men, however, the underlying mechanism shows a research gap.

Caputi *et al*. [50] conducted a study wherein they hypothesized that GM and critical components of the gut-brain axis influence the pain threshold in men and women, and they also conjectured that sex, different phases in the menstrual cycle and the use of contraceptive pills in women may be a major cause of the inter-sex pain differences. It was observed in their study that the pain tolerance threshold (PTT) and pain sensation threshold (PST) were higher in women compared to men, but the ratio of PTT/PST was significantly lower in women. Also, women undertaking contraceptive pills were associated with an increase in an abundance of certain bacterial genera which correlated positively with pain sensation thresholds. Therefore, it was concluded in their study that GM may be one of the factors determining inter-sex differences in pain perception.

## 5. Advances in the non-invasive therapeutic management of chronic orofacial and TMJ pain targeting gut dysbiosis

Chronic orofacial and TMJ pain is of complex and multifactorial aetiology that involves cross-over to other branches of dentistry and medicine thereby making the diagnosis and treatment cumbersome and challenging. The major interventions employed for treating chronic orofacial and TMJ pain involve psychological management like Cognitive Behavioural Therapy (CBT) [51], Acceptance and commitment therapy (ACT) [52], Pharmacological management [53], Lifestyle-based management [53] and Current-stimulation based management [53]. However, with evolving studies on the interlink between GM and chronic orofacial pain, the treatment should include a focus on targeting gut health to alleviate pain.

This section delves into the non-invasive treatment options to alter gut health and achieve effective chronic orofacial pain management (Fig. 3).

**Fig. 3.** Recent advances in non-invasive therapeutic options to treat gut dysbiosis which include (from clockwise)—probiotics, omega-3 fatty acid diet, resveratrol, microbiome engineering, good sleep cycle and lifestyle management. Created with BioRender.com.

### 5.1 Probiotics

A multitude of research is being carried out in the field of non-invasive therapeutics for chronic pain management, and probiotics are a recent addition to this list. Probiotics are also being used in the treatment of anxiety and depression [54, 55] and have shown ensuring results so much that, they are being referred to as “psychobiotics” [56]. They have been shown to modulate the reactivity of vast areas of the brain network in healthy women who consumed fermented dairy products for 4 weeks compared to those who consumed unfermented milk products [57].

There are multiple studies which have used probiotics for alleviating migraine-type headaches and the subjects have reported a reduction in the intensity, duration and frequency of headaches [58, 59, 60, 61]. Though the data on the mechanism of action of probiotics in reducing migraine-type headaches is unclear, it has been shown to increase butyrate production in the colon, which is reduced in migraineurs [62], improve gut permeability and attenuate inflammation [63].

### 5.2 Gluten-free diet

Dietary habits, lifestyle changes and sleep patterns are linked to exacerbating chronic pain. Gluten is one such dietary component that has been associated with gastrointestinal, neurologic, dermatologic, psychologic and musculoskeletal disorders [64]. In a 2021 study, done to evaluate the efficacy of a gluten-free diet (GFD) in chronic myofascial pain management of masticatory muscles in women, GFD seemed to reduce the pain sensitivity in women with Temporomandibular disorders (TMD) and increase the pressure pain threshold of Masseter and Anterior Temporalis muscle [65]. Though GFD may be used as an adjunctive therapy for chronic orofacial and TMD pain, further studies are needed.

### 5.3 Resveratrol

Resveratrol is a naturally occurring bioactive compound found mainly in grape skins and red wines, which has antioxidant and anti-inflammatory properties. It has been used in the treatment of trigeminal neuropathic pain in rats and chronic neuropathic pain in mice [34]. In a study performed to infer the effect of resveratrol on TMJ inflammation which was induced by complete Freund’s adjuvant (CFA) in mice, it was found that Resveratrol inhibits CFA-induced TMJ inflammation, reverses the CFA-induced reduction of short-chain fatty acids and restores the integrity of the blood-brain barrier. It was also found that there was a significant reduction of TMJ inflammatory pain with faecal microbiota transplantation (FMT) done using the faeces of Resveratrol-treated mice [34]. Hence, it was concluded in their study, that recovery of gut microbiota could be used as a promising therapeutic strategy for developing a new therapy for TMJ pain.

### 5.4 Omega-3 fatty acids

Dietary lipids like omega-3 fatty acids, play an important role in regulating gut health. Though there are multiple studies which showcase the effects of carbohydrates on host-specific gut microbiota, the impact of dietary lipids like omega-3 fatty acids still needs more research [66]. Omega-3 fatty acids help maintain intestinal wall integrity, improving the microbiota profiling and hence can be used as an adjunct to treat gut dysbiosis, neuropathic pain, depression, joint pain associated with rheumatoid arthritis and IBS [66].

### 5.5 Melatonin

Melatonin secreted by the pineal gland, is traditionally known for its role in a wide array of physiologic functions like regulating circadian rhythms, sleep and immune functions. However, its anti-inflammatory, nociceptive and antioxidant properties still need more research to validate. A 2015 study performed to study the effect of melatonin on acute pulpitis, discovered that acute pulpitis causes a reduction in serum melatonin levels and exogenous supplementation of melatonin alleviates pulpal pain [67]. Melatonin has also been proven to alter mechanical and thermal hyperalgesia induced by CFA in chronic orofacial pain model in rats, thus making it a potent antihyperalgesic [68]. Animal studies and even human clinical trials have shown promising results in treating myofascial TMJ pain, where patients introduced to melatonin showed a reduction in pain by 44% and increased pressure pain threshold by 39% compared to the placebo group [69]. Additionally, there is one documented report of melatonin used for the treatment of sleep-related bruxism in children [70].

### 5.6 Microbiome engineering

As the role of gut health dysbiosis and its link with human health and diseases is increasingly documented, genetically engineering the gut microbiota to diagnose, and treat autoimmune, metabolic, chronic pain and infectious diseases has overtaken the conventional methods of treating gut dysbiosis. This holistic approach helps us understand the relationship between gut microbiome and the host’s health. Clustered Regularly Interspaced Short Palindromic Repeats/CRISPR associated protein (CRISPR/Cas) systems, transposon-based systems, homologous recombination and integrase-based systems have been used as genome editing tools to culture the gut commensal bacteria. However, economical delivery and optimisation are still needed to harness their therapeutic potential and open new avenues for treating and managing multiple diseases [71].

## 6. Discussion

Chronic pain has become a leading cause of disability and amounts to enormous healthcare costs. The current therapies for treating orofacial and TMJ pain involve both invasive and non-invasive therapies. Even still, we do often come across patients who suffer from a consistent chronic type of pain with no relief from any model of treatment. Eventually, this type of chronic pain becomes adaptive and results in a negative sequela like Central sensitization [72].

Central sensitization results in pain hypersensitivity due to the amplification of neural signalling within the CNS and its features are documented in patients with fibromyalgia, rheumatoid arthritis, headache, osteoarthritis, *etc*. The prognosis of such cases is poor, and hence it is logical to shift our treatment option towards non-invasive form of precision pain medicine before any patient develops central sensitization due to persistent neural firing. Altering the gut microbiome to treat chronic diseases is a form of precision medicine which has become the new norm in treating multiple medical conditions. Despite the growing literature evidence on the interlink between GM and chronic types of orofacial pain, major studies are based on animal models and the literature lacks human trials.

The science and understanding of gut health and its vast association with the pathophysiology of multiple diseases have undergone substantial revision. There is rapid growth happening in the field of microbiome science, which has opened a new array of progress in the arena of the gut-brain axis.

## 7. Conclusions

From this review, it’s understood that the interlink between gut health and orofacial & TMJ pain is an area still unexplored despite its promising potential. The maxim “You are what you eat” finally stands as a testament to itself. This new perspective towards treating chronic orofacial and TMJ pain will help alleviate those with consistent chronic pain.

## References

[1] Cani PD. Human gut microbiome: hopes, threats and promises. Gut. 2018; 67: 1716–1725. 10.1136/gutjnl-2018-316723 PMC6109275 29934437

[2] Turnbaugh PJ, Ley RE, Hamady M, Fraser-Liggett CM, Knight R, Gordon JI. The human microbiome project. Nature. 2007; 449: 804–810. 10.1038/nature06244 PMC3709439 17943116

[3] Socała K, Doboszewska U, Szopa A, Serefko A, Włodarczyk M, Zielińska A, *et al*. The role of microbiota-gut-brain axis in neuropsychiatric and neurological disorders. Pharmacological Research. 2021; 172: 105840. 10.1016/j.phrs.2021.105840 34450312

[4] Quigley EMM. Microbiota-brain-gut axis and neurodegenerative diseases. Current Neurology and Neuroscience Reports. 2017; 17: 94. 10.1007/s11910-017-0802-6 29039142

[5] Chen J, Wang Q, Wang A, Lin Z. Structural and functional characterization of the GM in elderly women with migraine. Frontiers in Cellular and Infection Microbiology. 2020; 9: 470. 10.3389/fcimb.2019.00470 PMC7001586 32083024

[6] Harper DE, Schrepf A, Clauw DJ. Pain mechanisms and centralized pain in temporomandibular disorders. Journal of Dental Research. 2016; 95: 1102–1108. 10.1177/0022034516657070 PMC5004242 27422858

[7] Mayer EA, Nance K, Chen S. The gut-brain axis. Annual Review of Medicine. 2022; 73: 439–453. 10.1146/annurev-med-042320-014032 34669431

[8] Lassmann Ł, Pollis M, Żółtowska A, Manfredini D. Gut bless your pain-roles of the gm, sleep, and melatonin in chronic orofacial pain and depression. Biomedicines. 2022; 10: 1528. 10.3390/biomedicines10071528 PMC9313154 35884835

[9] Tang Y, Liu S, Shu H, Yanagisawa L, Tao F. GM dysbiosis enhances migraine-like pain via TNF-α upregulation. Molecular Neurobiology. 2020; 57: 461–468. 10.1007/s12035-019-01721-7 PMC6980505 31378003

[10] Bolaños MD, Zumba EG, Rodríguez ML. Burning mouth syndrome as a manifestation of an unbalanced psycho-neuro-immuno-endocrine axis in mentally ill women with intestinal dysbiosis: a literature review. World Journal of Advanced Research and Reviews. 2022; 14: 040–050.

[11] Li JS, Su SL, Xu Z, Zhao LH, Fan RY, Guo JM. Potential roles of GM and microbial metabolites in chronic inflammatory pain and the mechanisms of therapy drugs. Therapeutic Advances in Chronic Disease. 2022; 13: 20406223221091177. 10.1177/20406223221091177 PMC9340317 35924009

[12] Basbaum AI, Bautista DM, Scherrer G, Julius D. Cellular and molecular mechanisms of pain. Cell. 2009; 139: 267–284. 10.1016/j.cell.2009.09.028 PMC2852643 19837031

[13] Guo R, Chen LH, Xing C, Liu T. Pain regulation by GM: molecular mechanisms and therapeutic potential. British Journal of Anaesthesia. 2019; 123: 637–654. 10.1016/j.bja.2019.07.026 31551115

[14] Chiang CY, Sessle BJ, Dostrovsky JO. Role of astrocytes in pain. Neurochemical Research. 2012; 37: 2419–2431. 10.1007/s11064-012-0801-6 22638776

[15] Sessle BJ. Peripheral and central mechanisms of orofacial inflammatory pain. International Review of Neurobiology. 2011; 97: 179–206. 10.1016/B978-0-12-385198-7.00007-2 21708311

[16] Ji RR, Berta T, Nedergaard M. Glia and pain: is chronic pain a gliopathy? Pain. 2013; 154: S10–S28. 10.1016/j.pain.2013.06.022 PMC3858488 23792284

[17] Zhang ZJ, Jiang BC, Gao YJ. Chemokines in neuron-glial cell interaction and pathogenesis of neuropathic pain. Cellular and Molecular Life Sciences. 2017; 74: 3275–3291. 10.1007/s00018-017-2513-1 PMC11107618 28389721

[18] Donnelly CR, Andriessen AS, Chen G, Wang K, Jiang C, Maixner W, *et al*. Central nervous system targets: glial cell mechanisms in chronic pain. Cellular and Molecular Life Sciences. 2020; 17: 846–860. 10.1007/s13311-020-00905-7 PMC7609632 32820378

[19] Bradesi S, Svensson CI, Steinauer J, Pothoulakis C, Yaksh TL, Mayer EA. Role of spinal microglia in visceral hyperalgesia and NK1R up-regulation in a rat model of chronic stress. Gastroenterology. 2009; 136: 1339–1348. 10.1053/j.gastro.2008.12.044 PMC2812027 19249394

[20] Magni G, Riboldi B, Ceruti S. Modulation of glial cell functions by the Gut-Brain Axis: a role in neurodegenerative disorders and pain transmission. Cells. 2023; 12: 1612. 10.3390/cells12121612 PMC10297219 37371082

[21] Martins DO, Marques DP, Venega RAG, Chacur M. Photobiomodulation and B vitamins administration produces antinociception in an orofacial pain model through the modulation of glial cells and cytokines expression. Brain Behavior Immunity Health. 2020; 2: 100040. 10.1016/j.bbih.2020.100040 PMC8474295 34589831

[22] Zhang JD, Liu J, Zhu SW, Fang Y, Wang B, Jia Q, *et al*. Berberine alleviates visceral hypersensitivity in rats by altering gut microbiome and suppressing spinal microglial activation. Acta Pharmacologica Sinica. 2021; 42: 1821–1833. 10.1038/s41401-020-00601-4 PMC8563748 33558654

[23] Valesan LF, Da-Cas CD, Réus JC, Denardin ACS, Garanhani RR, Bonotto D, *et al*. Prevalence of temporomandibular joint disorders: a systematic review and meta-analysis. Clinical Oral Investigations. 2021; 25: 441–453. 10.1007/s00784-020-03710-w 33409693

[24] Munzenmaier DH, Wilentz J, Cowley AW III. Genetic, epigenetic, and mechanistic studies of temporomandibular disorders and overlapping pain conditions. Molecular Pain. 2014; 10: 72. 10.1186/1744-8069-10-72 PMC4269918 25511046

[25] Gallotta S, Bruno V, Catapano S, Mobilio N, Ciacci C, Iovino P. High risk of temporomandibular disorder in irritable bowel syndrome: is there a correlation with greater illness severity? World Journal of Gastroenterology. 2017; 23: 103–109. 10.3748/wjg.v23.i1.103 PMC5221272 28104985

[26] Versteeg RI, Serlie MJ, Kalsbeek A, la Fleur SE. Serotonin, a possible intermediate between disturbed circadian rhythms and metabolic disease. Neuroscience. 2015; 301: 155–167. 10.1016/j.neuroscience.2015.05.067 26047725

[27] Clarke G, Grenham S, Scully P, Fitzgerald P, Moloney RD, Shanahan F, *et al*. The Microbiome-Gut-Brain Axis during early life regulates the hippocampal serotonergic system in a sex-dependent manner. Molecular Psychiatry. 2013; 18: 666–673. 10.1038/mp.2012.77 22688187

[28] Ridaura V, Belkaid Y. GM: the link to your second brain. Cell. 2015; 161: 193–194. 10.1016/j.cell.2015.03.033 25860600

[29] Li Y, Fang M, Niu L, Fan Y, Liu Y, Long Y, *et al*. Associations among gastroesophageal reflux disease, mental disorders, sleep and chronic temporomandibular disorder: a case-control study. Canadian Medical Association Journal. 2019; 191: E909–E915. 10.1503/cmaj.181535 PMC6699946 31427355

[30] Zhou F, Wang X, Han B, Tang X, Liu R, Ji Q, *et al*. Short-chain fatty acids contribute to neuropathic pain via regulating microglia activation and polarization. Molecular Pain. 2021; 17: 1744806921996520. 10.1177/1744806921996520 PMC7925956 33626986

[31] Nagamine T. Burning mouth syndrome needs to consider the gut-brain axis from three types of pain: nociceptive, neuropathic, and nociplastic pain. Journal of Gastrointestinal and Liver Diseases. 2023; 32: 558–559. 10.15403/jgld-5322 38147602

[32] Lan Z, Wei Y, Yue K, He R, Jiang Z. Genetically predicted immune cells mediate the association between gut microbiota and neuropathy pain. Inflammopharmacology. 2024; 32: 3357–3373. 10.1007/s10787-024-01514-y PMC11416384 38955934

[33] Murakami N, Yoshikawa K, Tsukada K, Kamio N, Hayashi Y, Hitomi S, *et al*. Butyric acid modulates periodontal nociception in Porphyromonas gingivalis-induced periodontitis. Journal of Oral Science. 2022; 64: 91–94. 10.2334/josnusd.21-0483 34980829

[34] Ma Y, Liu S, Shu H, Crawford J, Xing Y, Tao F. Resveratrol alleviates temporomandibular joint inflammatory pain by recovering disturbed GM. Brain Behavior and Immunity. 2020; 87: 455–464. 10.1016/j.bbi.2020.01.016 PMC9444375 32001342

[35] Li XY, Yang CH, Lv JJ, Liu H, Zhang LY, Yin MY, *et al*. Global, regional, and national epidemiology of migraine and tension-type headache in youths and young adults aged 15–39 years from 1990 to 2019: findings from the global burden of disease study 2019. The Journal of Headache and Pain. 2023; 24: 126. 10.1186/s10194-023-01659-1 PMC10506184 37718436

[36] Jones R, Lydeard S. Irritable bowel syndrome in the general population. British Medical Journal. 1992; 304: 87–90. 10.1136/bmj.304.6819.87 PMC1880997 1737146

[37] Vandvik PO, Wilhelmsen I, Ihlebaek C, Farup PG. Comorbidity of irritable bowel syndrome in general practice: a striking feature with clinical implications. Alimentary Pharmacology & Therapeutics. 2004; 20: 1195–1203. 10.1111/j.1365-2036.2004.02250.x 15569123

[38] Peatfield RC, Glover V, Littlewood JT, Sandler M, Clifford Rose F. The prevalence of diet-induced migraine. Cephalalgia. 1984; 4: 179–183. 10.1046/j.1468-2982.1984.0403179.x 6498931

[39] van Hemert S, Breedveld AC, Rovers JM, Vermeiden JP, Witteman BJ, Smits MG, *et al*. Migraine associated with gastrointestinal disorders: review of the literature and clinical implications. Frontiers in Neurology. 2014; 5: 241. 10.3389/fneur.2014.00241 PMC4240046 25484876

[40] Aamodt AH, Stovner LJ, Hagen K, Zwart JA. Comorbidity of headache and gastrointestinal complaints. The Head-HUNT study. Cephalalgia. 2008; 28: 144–151. 10.1111/j.1468-2982.2007.01486.x 18197884

[41] Boyle R, Behan PO, Sutton JA. A correlation between severity of migraine and delayed gastric emptying measured by an epigastric impedance method. British Journal of Clinical Pharmacology. 1990; 30: 405–409. 10.1111/j.1365-2125.1990.tb03791.x PMC1368143 2223419

[42] Bates EA, Nikai T, Brennan KC, Fu YH, Charles AC, Basbaum AI, *et al*. Sumatriptan alleviates nitroglycerininduced mechanical and thermal allodynia in mice. Cephalalgia. 2010; 30: 170–178. 10.1111/j.1468-2982.2009.01864.x PMC4854191 19489890

[43] Tang Y, Liu S, Shu H, Xing Y, Tao F. AMPA receptor GluA1 Ser831 phosphorylation is critical for nitroglycerin-induced migraine- like pain. Neuropharmacology. 2018; 133: 462–469. 10.1016/j.neuropharm.2018.02.026 PMC5858972 29486167

[44] Mahmoudi J, Mohaddes G, Erfani M, Sadigh-Eteghad S, Karimi P, Rajabi M, *et al*. Cerebrolysin attenuates hyperalgesia, photophobia, and neuroinflammation in a nitroglycerin-induced migraine model in rats. Brain Research Bulletin. 2018; 140: 197–204. 10.1016/j.brainresbull.2018.05.008 29752991

[45] Perini F, D’Andrea G, Galloni E, Pignatelli F, Billo G, Alba S, *et al*. Plasma cytokine levels in migraineurs and controls. Headache 2005; 45: 926–931. 10.1111/j.1526-4610.2005.05135.x 15985111

[46] Sender R, Fuchs S, Milo R. Revised estimates for the number of human and bacteria cells in the body. PLOS Biology. 2016; 14: e1002533. 10.1371/journal.pbio.1002533 PMC4991899 27541692

[47] Yoon K, Kim N. Roles of sex hormones and gender in the gut microbiota. Journal of Neurogastroenterology and Motility. 2021; 27: 314–325. 10.5056/jnm20208 PMC8266488 33762473

[48] Mulak A, Taché Y, Larauche M. Sex hormones in the modulation of irritable bowel syndrome. World Journal of Gastroenterology. 2014; 20: 2433–2448. 10.3748/wjg.v20.i10.2433 PMC3949254 24627581

[49] Kwa M, Plottel CS, Blaser MJ, Adams S. The intestinal microbiome and estrogen receptor-positive female breast cancer. Journal of the National Cancer Institute. 2016; 108: djw029. 10.1093/jnci/djw029 PMC5017946 27107051

[50] Caputi V, Bastiaanssen TFS, Peterson V, Sajjad J, Murphy A, Stanton C, *et al*. Sex, pain, and the microbiome: the relationship between baseline gut microbiota composition, gender and somatic pain in healthy individuals. Brain Behavior and Immunity. 2022; 104: 191–204. 10.1016/j.bbi.2022.06.002 35688340

[51] Gore M, Sadosky A, Stacey BR, Tai KS, Leslie D. The burden of chronic low back pain: clinical comorbidities, treatment patterns, and health care costs in usual care settings. Spine. 2012; 37: E668–E677. 10.1097/BRS.0b013e318241e5de 22146287

[52] Hayes SC, Luoma JB, Bond FW, Masuda A, Lillis J. Acceptance and commitment therapy: model, processes and outcomes. Behaviour Research and Therapy. 2006; 44: 1–25. 10.1016/j.brat.2005.06.006 16300724

[53] Priyank H, Shankar Prasad R, Shivakumar S, Sayed Abdul N, Pathak A, Cervino G. Management protocols of chronic orofacial pain: a systematic review. Saudi Dental Journal. 2023; 35: 395–402. 10.1016/j.sdentj.2023.04.003 PMC10373074 37520608

[54] Desbonnet L, Garrett L, Clarke G, Kiely B, Cryan JF, Dinan TG. Effects of the probiotic bifidobacterium infantis in the maternal separation model of depression. Neuroscience. 2010; 170: 1179–1188. 10.1016/j.neuroscience.2010.08.005 20696216

[55] Liang S, Wang T, Hu X, Luo J, Li W, Wu X, *et al*. Administration of lactobacillus helveticus NS8 improves behavioral, cognitive, and biochemical aberrations caused by chronic restraint stress. Neuroscience. 2015; 310: 561–577. 10.1016/j.neuroscience.2015.09.033 26408987

[56] Müller N, Schwarz MJ. The immune-mediated alteration of serotonin and glutamate: towards an integrated view of depression. Molecular Psychiatry. 2007; 12: 988–1000. 10.1038/sj.mp.4002006 17457312

[57] Tillisch K, Labus J, Kilpatrick L, Jiang Z, Stains J, Ebrat B, *et al*. Consumption of fermented milk product with probiotic modu¬lates brain activity. Gastroenterology. 2013; 144: 1394–1401. 10.1053/j.gastro.2013.02.043 PMC3839572 23474283

[58] Sensenig J, Johnson M, Staverosky T. Treatment of migraine with targeted nutrition focused on improved assimilation and elimina¬tion. Alternative Medicine Review. 2001; 6: 488–494. 11703169

[59] de Roos NM, Giezenaar CG, Rovers JM, Witteman BJ, Smits MG, van Hemert S. The effects of the multispecies probiotic mix¬ture Ecologic®Barrier on migraine: results of an open-label pilot study. Beneficial Microbes. 2015; 6: 641–646. 10.3920/BM2015.0003 25869282

[60] de Roos NM, van Hemert S, Rovers JMP, Smits MG, Witteman BJM. The effects of a multispecies probiotic on migraine and markers of intestinal permeability-results of a randomized pla¬cebo-controlled study. European Journal of Clinical Nutrition. 2017; 71: 1455–1462. 10.1038/ejcn.2017.57 28537581

[61] Ghavami A, Khorvash F, Heidari Z, Khalesi S, Askari G. Effect of synbiotic supplementation on migraine characteristics and inflammatory biomarkers in women with migraine: results of a randomized controlled trial. Pharmacological Research. 2021; 169: 105668. 10.1016/j.phrs.2021.105668 33989763

[62] Moens F, Van den Abbeele P, Basit AW, Dodoo C, Chatterjee R, Smith B, *et al*. A four-strain probiotic exerts positive immuno¬modulatory effects by enhancing colonic butyrate production *in vitro*. International Journal of Pharmaceutics. 2019; 555: 1–10. 10.1016/j.ijpharm.2018.11.020 30445175

[63] Kiecka A, Szczepanik M. Migraine and the microbiota. Can probiotics be beneficial in its prevention?—A narrative review. Pharmacological Reports. 2024; 76: 251–262. 10.1007/s43440-024-00584-7 38502301

[64] Volta U, Bardella MT, Calabrò A, Troncone R, Corazza GR; Study Group for Non-Celiac Gluten Sensitivity. Study Group for non-celiac gluten sensitivity. An Italian prospective multicenter survey on patients suspected of having non-celiac gluten sensitivity. BMC Medicine. 2014; 12: 85. 10.1186/1741-7015-12-85 PMC4053283 24885375

[65] Araújo Oliveira Buosi J, Abreu Nogueira SM, Sousa MP, Soraya Costa Maia C, Regis RR, de Freitas Pontes KM, *et al*. Gluten-free diet reduces pain in women with myofascial pain in masticatory muscles: a preliminary randomized controlled trial. Journal of Oral & Facial Pain and Headache. 2021; 35: 199–207. 10.11607/ofph.2823 34609378

[66] Costantini L, Molinari R, Farinon B, Merendino N. Impact of Omega-3 fatty acids on the gut microbiota. International Journal of Molecular Sciences. 2017; 18: 2645. 10.3390/ijms18122645 PMC5751248 29215589

[67] Li JG, Lin JJ, Wang ZL, Cai WK, Wang PN, Jia Q,* et al*. Melatonin attenuates inflammation of acute pulpitis subjected to dental pulp injury. American Journal of Translational Research. 2015; 7: 66–78. PMC4346524 25755829

[68] Scarabelot VL, Medeiros LF, de Oliveira C, Adachi LN, de Macedo IC, Cioato SG, *et al*. Melatonin alters the mechanical and thermal hyperalgesia induced by orofacial pain model in rats. Inflammation. 2016; 39: 1649–1659. 10.1007/s10753-016-0399-y 27378529

[69] Vidor LP, Torres IL, Custódio de Souza IC, Fregni F, Caumo W. Analgesic and sedative effects of melatonin in temporomandibular disorders: a double-blind, randomized, parallel-group, placebo-controlled study. Journal of Pain and Symptom Management. 2013; 46: 422–432. 10.1016/j.jpainsymman.2012.08.019 23195393

[70] Erden S. Sleep-related bruxism response to melatonin Treatment. Journal of Child and Adolescent Psychopharmacology. 2020; 30: 201. 10.1089/cap.2019.0143 31800301

[71] Zheng L, Shen J, Chen R, Hu Y, Zhao W, Leung EL, *et al*. Genome engineering of the human gut microbiome. Journal of Genetics and Genomics. 2024; 51: 479–491. 10.1016/j.jgg.2024.01.002 38218395

[72] Nijs J, George SZ, Clauw DJ, Fernández-de-Las-Peñas C, Kosek E, Ickmans K, *et al*. Central sensitisation in chronic pain conditions: latest discoveries and their potential for precision medicine. The Lancet Rheumatology. 2021; 3: e383–e392. 10.1016/S2665-9913(21)00032-1 38279393

